# A Cross-Sectional Study on Sedentary Lifestyle Among Budding Doctors: Silent Killer?

**DOI:** 10.7759/cureus.53684

**Published:** 2024-02-06

**Authors:** Akhil R, Kajal Srivastava, Gayatri R Nair, Sai Mahesh Vajjala, Prerna Verma, Deepu Palal, Suman Ray

**Affiliations:** 1 Department of Community Medicine, Dr. D. Y. Patil Medical College, Hospital & Research Centre, Dr. D. Y. Patil Vidyapeeth (Deemed to be University), Pune, IND

**Keywords:** global physical activity questionnaire, exercise, neet, lifestyle, sedentary, interns, physical activity

## Abstract

Background

Internship is the time period when young doctors learn to balance between professional and personal lives. If they have good awareness and practice of any kind of physical activity during this period, it will help them to continue it forward. This enables them to educate and inspire people and patients around them. The main objectives of this study were to assess patterns of physical activity among medical interns and to understand the factors preventing physical activity among them.

Methodology

This cross-sectional survey was carried out among interns of a tertiary care hospital in western Maharashtra from July 2022 to September 2022. Ethical clearance was obtained before starting data collection. The survey was administered to those who fulfilled the inclusion criteria. The questionnaire was adopted from the Global Physical Activity Questionnaire. The data collected were entered into Microsoft Excel (Microsoft Corporation, Redmond, WA) and analysis was done using MedCalc v.18.2.1 (MedCalc Software Ltd, Ostend, Belgium).

Results

A total of 220 interns were enrolled in the survey, of which 13 were removed due to incomplete data and 28 interns did not participate in the study. Finally, 179 interns were included for analysis in the study. The response rate was 87.27%. The mean age of participants was 23.12 years. The study population consisted of 72 (40.22%) males and 107 (59.78%) females. Among participants, 33 interns (18.44%) were involved in vigorous activity during work, and 108 interns (60.34%) were involved in moderate physical activity during work. The median time of a sedentary lifestyle was 300 minutes per day and was more common among males. Cumulatively busy working schedules and exam preparation were the important reasons for reduced physical activity.

Conclusion

There is a gap in the practice of physical activity among interns. A sedentary lifestyle was more prevalent among male interns than in female interns. The main constraints were demanding working hours and the pressure of competitive exams. Medical students will become doctors in the future who can advise their patients on healthy lifestyle habits. We recommend that it is necessary to promote physical activity in medical schools and to reinforce the importance of physical exercise in the medical curriculum.

## Introduction

Around 31% of the world population aged 15 years or more engages in insufficient physical activity, and it contributes to the death of approximately 3.2 million people every year [1,2]. According to recent data, 80% of teenagers and 27% of adults do not engage in the WHO’s recommended level of physical activity [3].

A sedentary lifestyle is a risk factor for cardiovascular diseases (CVDs), diabetes mellitus (DM), hypertension (HTN), and cancers (breast, colon, colorectal, endometrial, and epithelial ovarian cancer) and increases the risk of mortality [4].

House surgeons or medical interns are the backbone of any medical college. They are the people who are involved in primary evaluation, diagnosis, treatment, and patient counselling. Most of the time due to increased working hours and duties, house surgeons might not be able to be involved in physical activities other than work-related activities. Studies have indicated that there is a lack of physical activity among medical students.

Internship is the time period when these young doctors learn to balance between professional and personal lives [5]. So, if they have a good awareness and practice of any kind of physical activity during this period, it will help them to continue it forward and educate and inspire people and patients around them.

In comparison to other streams of graduates, medical graduates have more knowledge regarding the need for physical activity [6]. But sometimes there is a gap in the attitude and practice of that knowledge.

One of the key components of community well-being is health promotion, which involves making changes to one's way of life, such as encouraging physical exercise and incorporating a balanced diet into one's daily activities. Health promotion activities are frequently conducted in medical schools as part of the curriculum. So medical students have a central role in health promotion [7].

It is essential for medical students to lead healthy lifestyles because they are more likely to deliver health promotional activities to the patients. Although it is generally believed that student behaviour is a temporary part of college life, sometimes disruptive behaviour at this level continues into adulthood. Therefore, university and college settings give us the chance to educate students on nutrition, health, and the need for a healthy lifestyle [6].

In this study, our objectives were to find the physical activity status among medical interns, the influence of parents and factors leading to reduced physical activity, and to provide recommendations to promote a healthy and active lifestyle.

## Materials and methods

A cross-sectional study was conducted among the medical interns of a tertiary care hospital in western Maharashtra from July 2022 to September 2022. All medical interns of the teaching hospital irrespective of their age, sex, past medical history, financial status, and physical parameters were included. Those who did not give informed written consent for the study were excluded.

Assuming the proportion of medical students practising physical activity from the study “Practice of physical activity among future doctors: a cross sectional analysis” [6] by Rao et al. as 61.9%, with an alpha of 5% and 80% power, at an accepted difference of 7%, the minimum sample size calculated was 185. The software used was WinPepi version 11.38.

The study was started after getting ethical clearance from the Institutional Ethics Sub-Committee, Dr. D. Y. Patil Medical College, Hospital & Research Centre (approval number: IESC/FP/18/2022). Medical interns doing their internship at the teaching medical hospital during the study period were enrolled for the assessment. We provided them with the participant information leaflet. The objectives and study methods were explained to them. After obtaining written informed consent from the participants, the World Health Organization's Global Physical Activity Questionnaire (GPAQ) was provided to them to be filled out via Google Forms (Google, Inc., Mountain View, CA) [8]. All interns posted in the same department were recruited together (4-40 interns at a time). Census sampling was used for gathering data. Once the questionnaire was filled and submitted, the data were entered into Microsoft Excel (Microsoft Corporation, Redmond, WA). Categorical data were presented in frequencies and percentages. Continuous data were presented as mean and standard deviation by assuming normality using the Shapiro-Francia test. Analysis was done with MedCalc statistical software version 18.2.1 (MedCalc Software Ltd, Ostend, Belgium; http://www.medcalc.org; 2018). P-value < 0.05 was considered statistically significant.

## Results

A total of 220 interns were enrolled in the survey, of which 13 were removed due to incomplete data and 28 interns did not participate in the survey. A total of 179 responses were included in the analysis. The response rate was 87.27%. The mean age of interns who participated in the study was 23.13 years with a standard deviation of 0.78. Among the participants, 107 (59.78%) were females and 72 (40.22%) were males. The mean BMI among males was 24.97 and among females, it was 23.49, who were in the overweight category according to the Asian standards (Table 1).

**Table 1:** Distribution of gender, BMI, and time spent reclined or sitting in a day among study participants. ^+^ The data have been represented as N (%). * The data have been represented as mean (SD). ^#^ The data have been represented as median (IQR).

Variable	Male	Female	Total
Gender^+^	72 (40.22)	107 (59.78)	179 (100)
Body mass index*	24.98 (3.81)	23.486 (3.63)	24.085 (3.77)
Time spent reclined or sitting (per day in minutes)^#^	330 (240-420)	300 (180-480)	300 (195-480)

In this survey, we found that only about one-third of male interns (22, 30.56%) and approximately half of female interns (50, 46.73%) were having normal BMI. The majority of male interns were obese (35, 48.61%), and around one-third (32, 29.91%) of females were in the obese category. Fourteen (19.44%) male and 18 (16.82%) female interns were overweight, and only one (1.39%) male and seven (6.54%) female interns were in underweight categories. The distribution of BMI among the male and female participants was statistically significant (Table 2).

**Table 2:** BMI category among participants. The data have been represented as N (%). * P-value < 0.05 was considered statistically significant.

Gender	Underweight	Normal	Overweight	Obese	Total	Significance
Male	1 (1.39)	22 (30.56)	14 (19.44)	35 (48.61)	72 (100)	Fisher's exact, P = 0.022*
Female	7 (6.54)	50 (46.73)	18 (16.82)	32 (29.91)	107 (100)	
Total	8 (4.47)	72 (40.22)	32 (17.88)	67 (37.43)	179 (100)	

Further, we explored the influence of the medical background of parents on the BMI of interns. We found that students with parents having medical backgrounds among normal, obese, overweight, and underweight categories were 22 (42.32%), 20 (38.46%), eight (3.85%), and two (3.85%), respectively. Students with non-medical backgrounds among normal, obese, overweight, and underweight categories were 50 (39.37%), 47 (37.01%), 24 (18.90%), and six (4.72%), respectively, but the distribution was not statistically significant (Table 3).

**Table 3:** Distribution of professional background of parents and BMI of students. The data have been represented as N (%). P-value < 0.05 was considered statistically significant.

	Underweight, n (%)	Normal, n (%)	Overweight, n (%)	Obese, n (%)	Total, n (%)	Significance
Parents in the medical profession	2 (3.85)	22 (42.31)	8 (15.38)	2 (3.85)	52 (100)	χ^2 ^= 0.42, df = 3, P = 0.94
Parents in the non-medical profession	6 (4.72)	50 (39.37)	24 (18.90)	6 (4.72)	127 (100)	
Total	8 (4.47)	72 (40.22)	32 (17.88)	67 (37.43)	179 (100)	

In this study, we explored the status of physical activity among participants and we observed that 33 (18.44 %) interns were involved in vigorous activity at work, which included activities that caused a large increase in heart rate or breathing, and 108 (60.34 %) of the interns were involved in moderate physical activity, which causes a small increase in heart rate or breathing (Table 4).

**Table 4:** Physical activity patterns among medical interns. The data have been represented as N (%).

Domains (n = 179)	Yes, N (%)
Vigorous activity at work	33 (18.44)
Moderate intensity at work	108 (60.34)
Vigorous recreational activity	65 (36.31)
Moderate recreational activity	64 (35.75)
Use of cycle or walk at least 10 minutes per day for daily commute	80 (44.69)

Further, we found out that 114 (63.69 %) interns were not involved in any kind of vigorous recreational activity during their leisure time. The vigorous recreational activity involves running, playing football, or other activities for at least 10 minutes a day. A similar trend was shown regarding moderate recreational activity as well. Only 64 (35.75%) interns were involved in moderate recreational activities like cycling, swimming, volleyball, or other activities at least 10 minutes a day during their leisure time. The survey revealed that 80 (55.31%) interns do not use bicycles or walk for 10 minutes at least per day for daily commutes. The median sedentary time spent sitting or in a reclined position during the entire day was 300 minutes, which was more common among male interns (330 minutes) (Table 1). Among all interns, 79 (44.13%) of them believed that they had adequate physical activity. From the remaining population of 179 interns, 53 (29.61%) participants pointed out a busy working schedule as the main reason for their reduced physical activity. National Eligibility cum Entrance Test postgraduate (NEET PG) preparation, lack of motivation, and other reasons like post-COVID, post-injury rehabilitation, and lack of space were revealed by 23 (12.85%), 19 (10.62%), and five (2.79%) participants, respectively (Table 5).

**Table 5:** Perceived reasons for reduced physical activity among medical interns. The data have been represented as N (%).

Perceived reasons for reduced physical activity (n = 179)	N (%)
No reduction in physical activity	79 (44.13)
Busy working schedule	53 (29.61)
National Eligibility cum Entrance Test postgraduate (NEET PG) preparation	23 (12.85)
No motivation	19 (10.62)
Others	5 (2.79)

## Discussion

Physical activity is an important factor that helps us to stay healthy and fit, which aids in leading a disease-free life. It should be a part of our lifestyle irrespective of occupation. It was proven that physical activity has positive effects on physical and mental health [9]. In the current study, the practice of physical activity by medical interns was low. Most of the interns did not meet the recommended physical activity guidelines laid out by the CDC [10]. This finding can be attributed to the busy schedules during internship and other academic obligations.

In a study done by Rao et al. in Karnataka, underweight, normal, overweight, and obese BMIs among male and female future doctors were 8.3% and 5.8%, 58.3% and 79.2%, 30.8% and 11.7%, and 2.5% and 3.3%, respectively [6], which was comparatively different from our findings. In the present study, underweight, normal, overweight, and obese BMIs among male and female future doctors were 1.39% and 6.54%, 30.56% and 46.73%, 19.44% and 16.82%, and 48.61% and 29.91%, respectively. Physical activity has a major role in maintaining a healthy BMI. This difference can be explained based on the variety of participants involved in the studies. The former study includes medical undergraduate students, who have plenty of free time out of academic schedules to carry out healthy lifestyles compared to medical interns in this current study who are expected to fulfil the round-the-clock medical services.

A study done by Nair et al. in South India points out that obesity and its burden among doctors can be attributed to a lack of physical activity [11]. Another reason for deranged BMI among adults and children is reduced or lack of sleep, which is applicable to medical interns who work round the clock in hospitals [12].

It was explained in studies that adult lifestyles and patterns are mostly driven by conscious choices, but it strongly depends on the socioeconomic status and the environment in which they reside [13]. Lower socioeconomic status is an established risk factor for obesity [14] but results from a population-based cohort study done in Norway revealed that the BMI of offspring is independent of parental education [15]. In this current study, we tried to explore the involvement of parental education, especially medical and non-medical background, on the BMI of their offspring and found that it did not have an impact.

In a study by Hemal Dave et al. [16] among the adolescent age group, it was reported that physical activity was relatively high within the male gender compared to the female gender and 69.3% of students were having lower levels of physical activity. Similarly, another study done by Joy et al. in Kerala among medical students found that 98 (54.44%) students were involved in a moderate level of physical activity and 30 (16.66%) had a high level of physical activity [17]. In the current study, we found that a sedentary lifestyle was more prevalent among male interns rather than in female interns. Moderate and vigorous levels of physical activity were found to be 60.34 % and 18.44%, respectively, in this study. Contrary to our finding, in a study done by Anand et al. in Delhi, the level of physical activity was low among medical students, and only 40% and 20% of males and females were physically active [18]. These differences can be attributed to the workload of interns in the respective departments and the type of lifestyle that they follow. It can be further influenced by the socio-economic class the participants belong to, the availability of recreational areas where they stay, and other personal interests.

Considering the factor of geographical variations in physical activity, according to a study by Anjana et al., the prevalence of physical inactivity in different states' urban settings is as follows, based on one of the largest phase-wise research done by the Indian Council for Medical Research on physical activity in different states of India: in all of these locations - Chandigarh (73%), Jharkhand (48%), Maharashtra (65%), and Tamil Nadu (71%), women demonstrated higher levels of physical inactivity than men [19]. Participants in the current study belong to different states in India, hence a physical inactivity of 39% among them can be explained on the basis of regional variations.

In a study done by Vidya et al., among medical students from Davangere, Karnataka, sedentary behaviour was considered if they spent sitting or reclining more than four hours in a typical day. More than 63% of the male students and 61.7% of the female students were following a sedentary lifestyle. Further, they evaluated the transportation domain by asking a question whether they walk or use a bicycle for at least 10 minutes continuously on a typical day for the day-to-day commute. Around 62% of the males and 75.7% of the females were fulfilling the criteria [7]. In the survey we conducted, the sedentary lifestyle was explored by asking the average time they spend sitting or reclined in a day. The median time spent sitting or reclining in a day was 300 minutes, which is almost five hours in a day, apart from the time they spend sleeping at night. Males spent a median of 330 minutes and females spent a median of 300 minutes on a normal day without any physical activity. Not only that, the proportion of the interns using a bicycle or walking for at least 10 minutes in a day for daily commute was 44.69%. This level of physical inactivity is concerning, especially in an era where non-communicable lifestyle diseases are rising.

As we discussed earlier, reasons for reduced physical activity can vary from person to person. In the survey, we encouraged our interns to explain the reasons for their reduced level of physical activity. The main reasons that came across were busy working schedules (29.61%), NEET PG entrance exam preparations (12.85%), and lack of motivation (10.61%). Other reasons explained were post-injury rehabilitation, post-COVID health phase, and lack of space for exercising. In a study with similar objectives by Rao et al. from Karnataka, the reasons mainly pointed out by students as hindering factors for not exercising were lack of time (60.5%), laziness (61.8%), and burnout due to college activities (42%) [6]. In another study from Kerala, when asked about what they consider obstacles to physical activity among students, 69% of them said they do not have enough time to exercise, 44% said they lack the motivation to engage in physical activity, and >15% said they found it inconvenient to exercise [17]. One of the main reasons we can observe in all the studies are lack of time or busy working schedules and a lack of motivation among young doctors. This sheds light on hectic working hours in teaching hospitals, the need for duty offs, fixed maximum working hours per week, and a healthy environment encouraging an active physical life.

In this current study, we were able to point out the lack of practice of physical activity among interns. Parental education and their influence over physical activity showed no association, hence we can deduce that behavioural modifications should target more on younger population. Further, the study revealed the current deterrents in the practice of physical activity like post-COVID, pressure of competitive exams, lack of designated areas for recreation and sports, etc. Hence, we believe that this study can give strength to current discussions on the need for curriculum and policy changes. The main limitation we faced during the study is that internship duties and schedules vary from department to department. Hence that can affect the physical activity patterns, but we have not taken it into consideration. The study was limited to budding doctors in a small geographical area and an urban teaching hospital setting due to practicality issues. Another important limitation is that we did not utilize more reliable electronic devices to evaluate the physical activity among the study participants, which may help to draw more accurate conclusions. Further evaluations on a larger population and multiple locations with the utilization of electronic devices to monitor physical activity will provide more light into the patterns and practices of physical activity of medical interns.

## Conclusions

There is a reluctance in the practice of physical activity among interns. The majority of male interns had abnormal BMIs. There was no influence of parental education on students’ insufficient physical activity levels. More than half of the interns were involved in moderate-intensity activities during work, and the majority of the participants were not involved in either vigorous or moderate recreational physical activity. A sedentary lifestyle is more prevalent among male interns. The average time spent sitting or inclined is around five hours per day. The main constraints of reduced physical activity are demanding working hours and the pressure of competitive exams. We recommend that to prepare medical students to become doctors of the future, who can advise their patients on healthy lifestyle habits, it is necessary to promote physical activity in medical schools and to reinforce the importance of physical exercise in the medical curriculum. The current competency-based medical education curriculum has included yoga as a part of routine education. Further research on the impact of this step will help us formulate and implement further changes. Measures to confirm decent working hours and avoid a toxic work culture should be implemented. A longitudinal study that observes students over their academic careers, across various educational groups and a range of disciplines will add value and weight to the characteristics that encourage students to engage in physical exercise.
